# Comparing the effectiveness and safety between triple antiplatelet therapy and dual antiplatelet therapy in type 2 diabetes mellitus patients after coronary stents implantation: a systematic review and meta-analysis of randomized controlled trials

**DOI:** 10.1186/s12872-015-0114-1

**Published:** 2015-10-09

**Authors:** Pravesh Kumar Bundhun, Tao Qin, Meng-Hua Chen

**Affiliations:** Institute of Cardiovascular Diseases, the First Affiliated Hospital of Guangxi Medical University, Nanning, Guangxi 530027 P. R. China

**Keywords:** Antiplatelet therapy, Cilostazol, Type 2 diabetes mellitus, Coronary stents implantation

## Abstract

**Background:**

Since antiplatelet therapy in type 2 diabetes mellitus (T2DM) patients is very important after intracoronary stenting, and because the most commonly used therapies have been the dual antiplatelet therapy (DAPT) consisting of aspirin and clopidogrel and the triple antiplatelet therapy (TAPT) consisting of aspirin, clopidogrel and cilostazol, we aim to compare the effectiveness and safety between triple antiplatelet therapy and dual antiplatelet therapy in T2DM patients.

**Methods:**

Systematic literature search was done from the databases of PubMed, Cochrane, Embase, China National Knowledge Infrastructure (CNKI) and WanFang. Randomized controlled trials (RCTs) comparing the effectiveness and safety between triple therapy and dual therapy in T2DM patients after coronary stents placement were included. Endpoints included major adverse cardiac effects (MACEs), target lesion revascularization (TLR), target vessel revascularization (TVR), death, stent thrombosis, bleeding and adverse drug reactions during a 9–12 months period, as well as platelet activities.

**Results:**

Four studies including 1005 patients reporting the adverse clinical outcomes and six studies including 519 patients reporting the platelet activities, with a total of 1524 patients have been analyzed in this meta-analysis. The pooling analysis shows that TAPT has significantly decreased the occurrence of MACEs (RR: 0.55; 95 % CI: 0.36–0.86, *P* = 0.009), TLR (RR 0.41; 95 % CI: 0.21–0.80, *P* = 0.008), TVR (RR 0.55; 95 % CI: 0.34–0.88, *P* = 0.01) and the overall incidence of Death/ Myocardial Infarction (MI)/TVR (RR 0.54; 95 % CI: 0.31–0.94, *P* = 0.03) during this 9 to 12 months follow up period after stents implantation. Stent thrombosis was almost similar in both groups. Bleeding seemed to favor DAPT but the result was not statistically significant. Platelet aggregation, platelet reactivity index (PRI) and platelet reactivity unit (PRU) were also reduced with Weight Mean Difference (WMD) of (−13.80; 95 % CI: −17.03 to −10.56, *P* < 0.00001), (−22.87; 95 % CI: −23.66 to −22.07, *P* < 0.00001) and (−44.17; 95 % CI: −58.56 to −29.77, *P* < 0.00001) respectively.

**Conclusion:**

Since MACEs have been significantly decreased in the triple group, TAPT appears to be more effective than DAPT in T2DM patients after intracoronary stenting. No significant difference in stent thrombosis and bleeding risks between these 2 groups shows TAPT to be almost as safe as DAPT in these diabetic patients.

## Background

Nowadays percutaneous coronary intervention (PCI) is becoming the preferred invasive procedure in patients suffering from coronary artery disease (CAD) and acute coronary syndrome (ACS) which are chronic macrovascular complications in type 2 diabetes mellitus (T2DM) patients. Several studies have shown that T2DM patients are more exposed to many adverse clinical outcomes such as major adverse cardiac effects (MACEs), target lesion revascularization (TLR), target vessel revascularization (TVR) and stent thrombosis after intracoronary stenting [1]. Platelet dysfunction is among one of the reasons T2DM patients have an increased risk of athero-thrombotic events [2, 3]. In particular, T2DM patients have increased platelet reactivity warranting the use of platelet-inhibiting strategies in order to reduce their ischemic risk after stents implantation. Since cardiovascular disease is the leading cause of morbidity and mortality in patients with T2DM and because the latter usually present with atypical symptoms in these patients, antiplatelet therapies in these high risk patients after coronary stents placement are extremely important. Although currently approved antiplatelet treatment strategies have proven useful in improving outcomes, T2DM patients continue to have a higher risk of adverse cardiovascular events especially after stents implantation. Several antiplatelet therapies have been tried but the most commonly used have been the dual antiplatelet therapy (DAPT) consisting of aspirin and clopidogrel, as mentioned in Emmanouil’s study [4], and the triple antiplatelet therapy (TAPT) consisting of aspirin, clopidogrel and cilostazol [5–9]. Studies show that TAPT has been a better option in T2DM patients compared to DAPT [10] but the treatments have not yet been well established systematically. Therefore, by performing a meta-analysis, we sought to evaluate systematically whether TAPT is more effective and safe to use compared to DAPT in T2DM patients after coronary stents implantation.

The abbreviations and acronyms used in this meta-analysis have been represented in Table 1.

**Table 1:** Abbreviations and acronyms

T2DM	Type 2 diabetes mellitus
PCI	Percutaneous coronary intervention
DAPT	Dual antiplatelet therapy
TAPT	Triple antiplatelet therapy
TVR	Target vessel revascularization
TLR	Target lumen revascularization
MACEs	Major adverse cardiac effects
PRI	Platelet reactivity index
WMD	Weight mean difference

## Methods

### Eligibility and search strategy

Relevant randomized controlled trials (RCTs) were identified through a computerized literature search from PubMed, Cochrane, Embase/Science Direct, China National Knowledge Infrastructure (CNKI) and WanFang databases using the search terms “Diabetes + Coronary Stents + Dual and/or Triple Anti platelet therapy”. Different wordings of the same meanings have also been used such as “PTCA/PCI, DAPT, TAPT, T2DM, coronary angioplasty and anti-platelets”. No language restrictions were used. All data were independently extracted by two investigators and then the results were compared, and disagreements were resolved by discussing with a third investigator before December 2014.

### Inclusion and exclusion criteria

Studies included in our meta-analysis were trials that: (a) randomly assigned patients for RCTs (b) included T2DM patients (wherever possible, selecting only T2DM patients in studies consisting of both T2DM and non-T2DM or retrieving the number of diabetic patients among other disease conditions such as acute coronary syndrome or long coronary lesion) (c) compared TAPT (aspirin, clopidogrel and cilostazol) with DAPT (aspirin and clopidogrel) (d) reported any of the corresponding endpoints including MACEs, TLR, TVR, stent thrombosis, bleeding and adverse drug reactions as well as platelet aggregation, PRI and PRU.

Exclusion criteria were trials (a) of retrospective non-randomized studies (b) including only non-diabetic patients (c) including another drug instead of cilostazol in the TAPT group (d) considering only DAPT or TAPT separately without comparison (e) with duplicate reports.

### Data extraction and quality assessment

Two authors (P.K.B. and T.Q.) independently reviewed the data and assessed the eligibility and methodological quality of each eligible trial. Information regarding study and patient characteristics, intervention strategies, and the pre-specified clinical outcomes was systematically extracted. Disagreements were discussed between the authors, and if the authors could not reach a consensus, disagreements were resolved by the third author (M.H.C.). The bias risk of trials was assessed with the components recommended by the Cochrane Collaboration, including sequence generation of the allocation, allocation concealment, blinding of participants, personnel, and outcome assessors, incomplete outcome data, selective outcome reporting, and other sources of bias. Quality scale was used to assess the trials: (A) true randomization and allocation concealed, and (B) process of randomization not given and concealment of allocation unclear. This approach was recommended by Cochrane Collaboration.

### Definitions, outcomes and follow-up

*Major adverse cardiac effects*: MACEs were defined as death of cardiac or procedure-related origin, MI, and repeat target lesion revascularization after stents implantation. Major adverse cardiovascular and cerebrovascular events (MACCEs) have also been considered together in this section.

*Revascularization*: included TLR and TVR. Revascularization was clinically indicated if there was >70 % diameter stenosis on angiography or >50 % stenosis together with a positive stress test or ischemic symptoms. TLR was defined as clinically indicated percutaneous or surgical revascularization of the index lesion during follow-up.

*Stent thrombosis*: which could occur acutely (within 24 h), sub-acutely (within 30 days), or as late as 1 year (late) after stent placement throughout the whole follow up period.

*Bleeding*: was defined as any hemorrhagic event which could occur with the use of the anti-platelets after stents implantation and included minor, major or minimal bleeding in this study.

Adverse drug reactions: included the adverse effects associated with the use of these anti-platelets. For example, rashes, gastrointestinal troubles, thrombocytopenia, liver dysfunction and finally drug discontinuation due to these adverse effects.

*Platelet aggregation*: defined as the clumping together of platelets in the blood.

*PRI and PRU*: dealt with the reactivity and activation of platelets after antiplatelet use.

The follow-up for these adverse clinical outcomes as well as the adverse drug reactions was for a period of 9 months in two studies and 12 months in the other two studies. For the platelet aggregation, PRI and PRU, the follow up period was randomly assumed to be during any given time after stents implantation.

### Statistical analysis

Risk ratio (RR) with 95 % confidence interval (CI) was used to express the pooled effect on discontinuous variables. For continuous variables, standard deviation for each group was calculated using the formula $s=\sqrt{\frac{1}{N-1}{\displaystyle \sum_{i=1}^N{\left({x}_i-\overline{x}\right)}^2}}$ and data were evaluated by means of weighted mean differences (WMDs) and 95 % confidence intervals (CIs). Heterogeneity across the trials was assessed using the Cochrane Q-statistic (*p* ≤0.05 was considered significant) and I^2^-statistic. I^2^ describes the percentage of total variation across studies that is due to heterogeneity rather than chance. A value of 0 % indicates no heterogeneity, and larger values indicate increased heterogeneity. If I^2^ was <50 %, fixed effect model was used. However, if I^2^ was >50 %, a random effect model was used [11]. Publication bias was visually estimated by assessing funnel plots. The pooled analyses were performed with RevMan 5.3.

## Results

### Selected studies and baseline characteristics

From the initial literature search, 211 items in PubMed, 597 in Embase, 341 in CNKI, 416 in WanFang databases and 19 in the Cochrane database were identified. After an elaborative screening, 15 RCTs satisfied our inclusion criteria studies. However, after eliminating the duplicate studies, 10 RCTs have been finally selected for our meta-analysis.

**Fig. 1:**
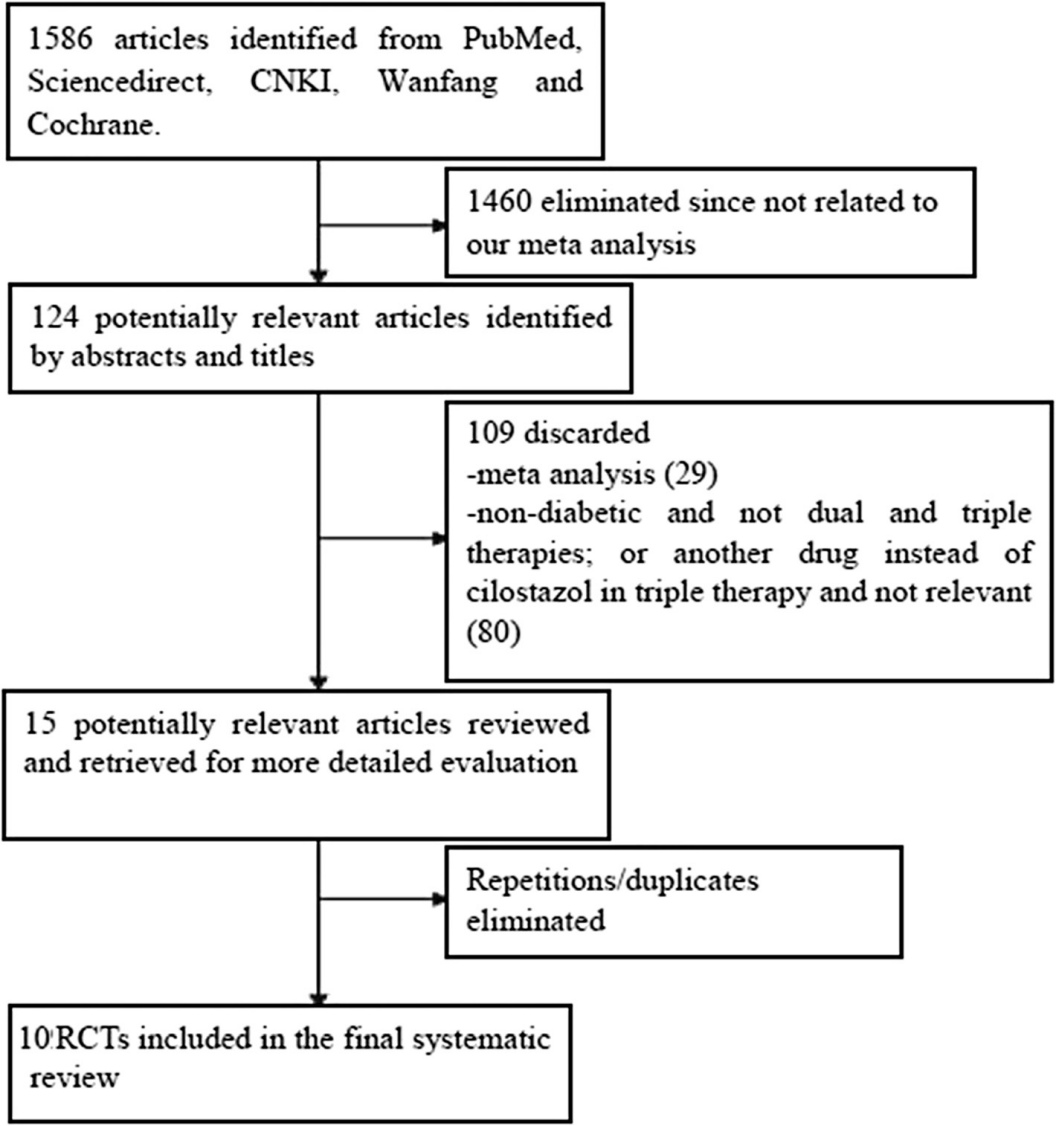
Flow diagram for the study selection. One thousand five hundred eighty six articles were identified from PubMed, Embase/Science Direct, CNKI, Wanfang and Cochrane databases. After considering the inclusion and exclusion criteria, finally 8 RCTs were selected and included in this meta-analysis. Showing the detailed flow chart of study selection

Among these ten studies, four studies including 1005 patients (487 in the DAPT, 518 in the TAPT group) reported the adverse clinical outcomes in these T2DM patients whereas the remaining six studies with 519 patients (287 in the DAPT group, 232 in the TAPT group) reported the platelet activities. A total of 1524 patients (774 and 750 patients following TAPT and DAPT respectively) fulfilled the inclusion criteria and have been finally included in our meta-analysis. Figure 1 shows the flow of the process for identifying potentially eligible trials and the reasons for exclusion.

Trials included in this meta-analysis with their total number of patients (including DAPT and TAPT) and their corresponding endpoints have been represented in Table 2.

**Table 2:** Shows the endpoints reported by each of the included trials

Trials	No of patients (DAPT + TAPT)	Follow up period (month)	Reported endpoints/outcomes
Han 2009 [6]	263	12	MACEs, TVR, and ADR.
Lee 2011 [33]	176	12	MACEs, TVR, TLR, ST, D/MI/TVR, BL and ADR.
Lee 2008 [5]	400	9	MACEs, TVR, TLR, ST, D/MI/TVR, BL and ADR.
Lee 2007 [34]	166	9	MACEs, TVR, TLR, ST, D/MI/TVR, BL and ADR.
Yang 2011 [35]	154	–	PRU and PA
Yang 2007 [36]	55	7	PA
Capranzano 2012 [37]	80	1	PRU and PRI
Angiolillo 2008 [38]	40	1	PRI and PA
Angiolillo 2011 [39]	106	1	PRI
Ha 2013 [40]	84	2	PRU and PA

*Abbreviations*: *MACEs* major adverse cardiac effects, *TVR* target vessel revascularization, *TLR* target lesion revascularization, *BL* bleeding, *ST* stent thrombosis, *ADR* adverse drug reactions, *D* death, *MI* myocardial infarction, *PA* platelet aggregation, *PRI* platelet reactivity index, *PRU* platelet reactivity unit

According to Table 2, four studies reported adverse cardiovascular outcomes as their endpoints. The other remaining trials dealt with platelet activities.

Table 3 summarizes the baseline characteristics for each of the included trials. No significant differences have been found in the baseline characteristics between the 2 groups.

**Table 3:** Baseline characteristics of patients from the included studies

Study/Year	Age (yrs)	Male (%)	Ht (%)	Hc (%)	Cs (%)	Q^a^
	T/D	T/D	T/D	T/D	T/D	
Lee 2007 [34]	60.9/61.2	64.8/63.6	54.8/55.2	30.0/28.4	37.6/37.2	B
Lee 2008 [5]	61.0/60.7	59.0/57.0	59.5/59.5	30.5/28.5	24.0/31.5	B
Yang 2011 [35]	63.5/63.5	62.3/62.3	55.8/55.8	37.7/37.7	28.6/28.6	B
Yang 2009 [36]	63.4/63.8	40.0/60.0	61.9/57.1	28.6/41.2	14.3/20.6	B
Capranzano 2012 [37]	60.4/60.4	60.2/60.2	91.2/91.2	97.0/97.0	37.1/37.1	B
Angiolillo 2008 [38]	64.0/64.0	60.0/60.0	96.0/96.0	92.0/92.0	24.0/24.0	B
Angiolillo 2011 [39]	60.5/60.5	63.0/63.0	95.0/95.0	100.0/100.0	28.0/28.0	B
Ha 2013 [40]	64.9/62.3	67.7/76.1	71.4/76.1	33.3/42.5	23.8/23.8	B
Lee 2011 [33]	60.9/62.1	70.0/71.5	58.4/64.7	42.4/45.0	30.4/30.1	B
Han 2009 [6]	59.6/60.2	73.8/72.9	57.9/56.1	45.5/45.4	–	B

*Abbreviations*:*NM* not mentioned, *Ht* hypertension, *Hc* hypercholesterolemia; *Cs* current smoker, *T* triple therapy, *D* dual therapy

^a^Quality scale: A:true randomization and allocation concealed; B:process of randomization not given and concealment of allocation unclear

Patients in most of the studies used beta-blockers and ACEI/ARB routinely. Moreover, patients in few of the studies also use statins as part of their routine medications. The use of glycoprotein IIb/IIIa was dependent on the physician’s decision during the procedure.

For the studies related to platelet activities, such as platelet aggregation, PRI and PRU; the mean has been calculated first as well as the standard deviation. In a few of the studies, since there was a period of crossover adding cilostazol to the DAPT or removing cilostazol from the TAPT after complete washout; the participants have been considered as different individuals before and after the crossover period despite of being the same patients. For example, in Angiolillo’s study there were 20 participants. These 20 participants were treated first with DAPT for 2 weeks and then after a washout period, the same 20 participants were treated with TAPT. In such a case, we have considered the number of patients undergoing DAPT to be 20 and the number of patients undergoing TAPT to be 20. And finally we have considered the total number of patients to be 40. These values have been used in the results.

All the patients were above 60 years old of age and most of them were males. In three studies, the percentage of patients suffering from hypertension was above 90 % while in the remaining studies, 50–70 % suffered from high blood pressure. Three studies reported a hyperlipidemia level of more than 90 % whereas the amount of participants who were smokers did not exceed 40 %.

The mean Quantitative Angiographic Measurement of the included studies showing the lesion length, stented length, minimal lumen diameter before and after the procedure and the diameter of the stenosis before and after the procedure have been illustrated in Table 4.

**Table 4:** Shows the mean Quantitative Angiographic Measurement of the included studies^a^

Variables (mm)	TAPT group (*n* = 377)	DAPT group (*n* = 365)
Reference diameter	2.81	2.79
Lesion length	31.5	31.7
Stented length	37.45	36.05
Minimal lumen diameter in segment before procedure	0.81	0.79
Minimal lumen diameter in segment after procedure	2.25	2.24
Minimal lumen diameter in stent after procedure	2.57	2.58
Stenosis diameter in segment before procedure (%)	70.3	70.4
Stenosis diameter in segment after procedure (%)	17.7	17.2
Stenosis diameter in stent after procedure (%)	8.45	7.57
Acute gain in segment	1.44	1.46
Acute gain in stent	1.76	1.79

^a^Data from trials Lee 2007 [34], Lee 2008 [5] and Lee 2011 [33] have been included in this table. Quantitative Angiographic Measurement data for study Han 2009 [6] was not available and therefore have been omitted

There were no differences in angiographic measurements between the 2 groups in those included studies.

### Result of the meta-analysis

The pooling analysis shows that TAPT significantly decreased the occurrence of overall MACEs among the 1005 patients reporting this outcome with (RR: 0.55, 95 % CI: 0.36–0.86; *P* = 0.009), TLR (RR 0.41; 95 % CI: 0.21–0.80; *P* = 0.008) and TVR (RR 0.55; 95 % CI: 0.34–0.88; *P* = 0.01) in these T2DM patients after coronary stents implantation. TAPT treatment showed superiority in reducing the overall Death/MI/TVR (RR 0.54; 95 % CI: 0.31–0.94; *P* = 0.03) compared to DAPT. Stent thrombosis (RR 0.95; 95 % CI: 0.19–4.76, *P* = 0.95) was similar in both groups and the result for bleeding (RR 0.80; 95 % CI: 0.40–1.59, *P* = 0.52) was not statistically significant. TAPT showed to be more beneficial in these T2DM patients by significantly reducing the platelet aggregation WMD: (−13.80; 95 % CI: −17.03 to −10.56, *P* < 0.00001), PRI WMD: (−22.87; 95 % CI: −23.66 to −22.07, *P* < 0.00001) and PRU WMD: (−44.17; 95 % CI: −58.56 to −29.77, *P* < 0.00001) respectively.

**Fig. 2:**
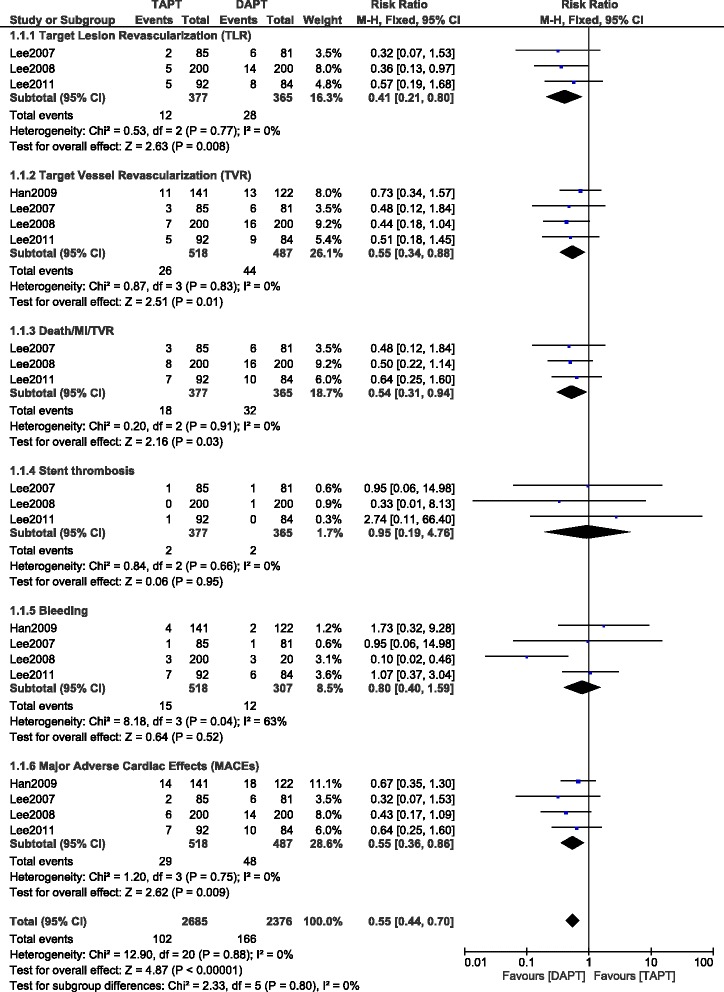
Forest plot comparing the adverse clinical outcomes between TAPT and DAPT in T2DM patients after intracoronary stenting. Adverse clinical outcomes have decreased with the use of this TAPT during a follow up period of 9 to 12 months after coronary stenting

**Fig. 3:**
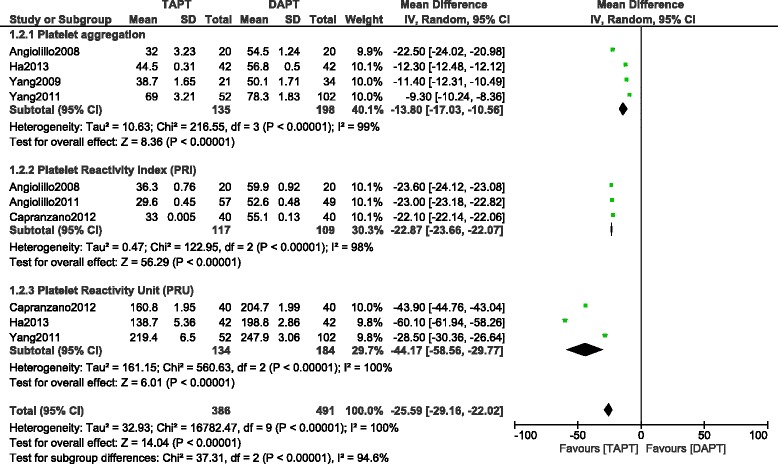
Forest plot comparing the platelet activities between TAPT and DAPT in T2DM after intracoronary stenting. A lower platelet aggregation, platelet reactivity index and platelet reactivity unit in the TAPT group shows that TAPT seems to be more effective in these T2DM patients

**Fig. 4:**
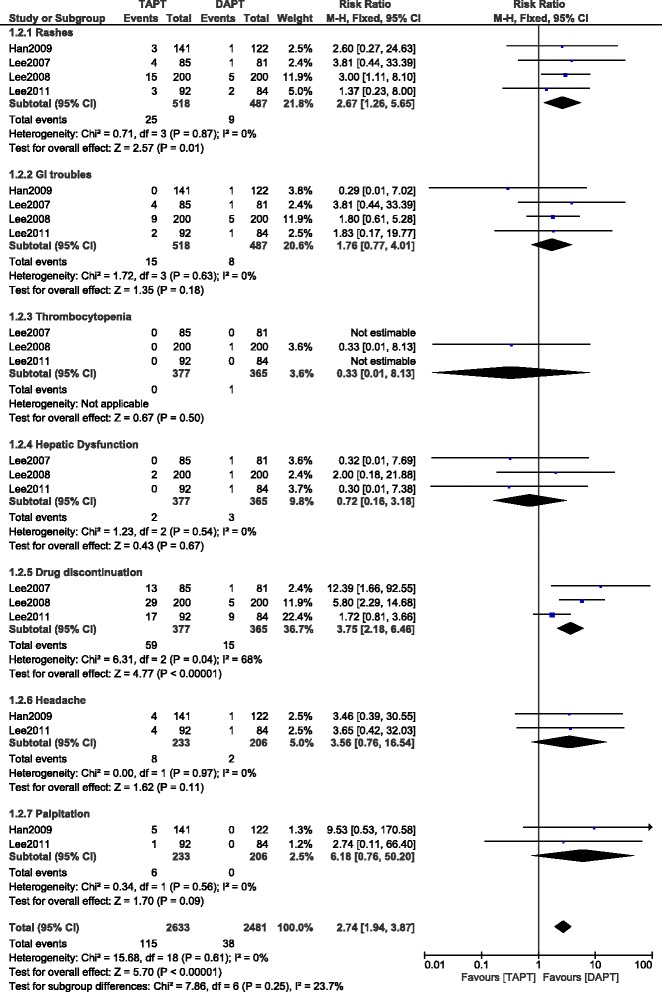
Forest plot comparing the adverse drug reactions between TAPT and DAPT in T2DM patients after intracoronary stenting. More adverse effects are associated with cilostazol use in this TAPT compared to DAPT in these T2DM patients

Results for the associated adverse effects between these 2 groups were as follow: (RR 2.67; 95 % CI: 1.26–5.65; *P* = 0.01) for rashes being higher in the TAPT group; (RR 1.76; 95 % CI: 0.77–4.01; *P* = 0.18) for gastrointestinal troubles; (RR 0.33; 95 % CI: 0.01–8.13; *P* = 0.50) for thrombocytopenia; (RR 0.72; 95 % CI: 0.16–3.18; *P* = 0.67) for hepatic dysfunction; (RR 6.18; 95 % CI: 0.76–50.20; *P* = 0.09) for palpitation and (RR 3.56; 95 % CI: 0.76–16.54; *P* = 0.11) for headache. Moreover, the risk ratio for patients to discontinue TAPT due to these adverse effects was (RR 3.75; 95 % CI: 2.18–6.46; *P* < 0.00001). The detailed results have been illustrated in Figs. 2, 3 and 4.

**Fig. 5:**
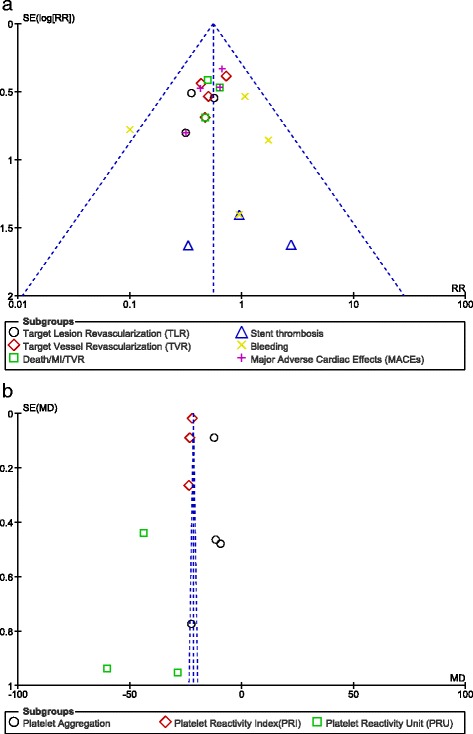
**a** and **b** Funnel plots assessing publication bias in the included studies. For all of the above analyses, sensitivity analyses yielded consistent results. Based on a visual inspection of the funnel plots, there has been no evidence of publication bias for the included studies that assessed all clinical end points

The funnel plots assessing publication bias in the included studies have been illustrated in Fig. 5a and b.

For all of the above analyses, sensitivity analyses yielded consistent results. Based on a visual inspection of the funnel plots, there has been no evidence of publication bias for the included studies that assessed all clinical endpoints.

## Discussion

### Aim and results of this study

The aim of the current study was to perform a comprehensive meta-analysis of several RCTs comparing and analyzing the effectiveness and safety between TAPT (aspirin, clopidogrel and cilostazol) and DAPT (aspirin and clopidogrel) in T2DM patients after coronary stents implantation.

Results from this study including 1005 patients reporting adverse clinical outcomes as their endpoints show that TAPT is more effective than DAPT in several aspects in T2DM patients. TAPT has significantly decreased platelet aggregation, PRI and PRU among the 519 patients analyzed. MACEs, TLR and TVR which are more prominent among diabetic patients, have also decreased significantly with the use of this cilostazol based TAPT. Stent thrombosis was similar in both groups. However, TAPT did not significantly increase the bleeding in these T2DM patients.

### Mechanisms of cilostazol in this TAPT

Several reasons are associated with these benefits and effectiveness of this TAPT. First of all, cilostazol has a different mechanism of action compared to aspirin and clopidogrel. It is a phosphodiesterase III (PDE3) inhibitor that plays the role of increasing cyclic adenosine monophosphate within platelets and thus leads to reduced aggregation of platelets [12]. Cilostazol can also inhibit expression of stent-induced P-selectin and the subsequent Mac-1–mediated leukocyte activation that is the trigger of restenosis after PCI [13]. To further explain this mechanism is thought to be the inhibition of neo-intimal proliferation, considered to be a major mechanism of restenosis after PCI caused by smooth muscle cells (SMC) migration, proliferation, and matrix synthesis. SMC migration and proliferation are induced by growth factors released from activated platelets. As an antiplatelet medication, cilostazol controls the induction by platelet-derived growth factors. More importantly, cilostazol is thought to directly inhibit SMC growth. In addition, there is much evidence that Mac-1 is one of the key proteins in the mechanism of restenosis. Studies have demonstrated clinically that PCI induced activation and upregulation of Mac-1 on the surface of neutrophils and that Mac-1 kinetics were linked to angiographic late lumen loss—known as neo-intimal thickening. Cilostazol may inhibit Mac-1–mediated leukocyte activation (act as a Mac-1 blocker) directly or through P-selectin–mediated platelet activation, which may lead to a reduction in the rate of restenosis after coronary stent implantation. This unique property of cilostazol makes it different from all the other anti-platelet agents.

Moreover, since T2DM have platelets hyperactivity, and are usually victims of aspirin and clopidogrel hypo-responsiveness, this adjunctive cilostazol can help to inhibit platelet aggregations and activation which might not be completely possible with the use of DAPT [14]. These mechanisms could be used to explain the better effect of this cilostazol-based TAPT compared to DAPT especially in these T2DM patients who are more at risk of these serious adverse cardiac events after coronary stenting.

### TAPT and DAPT in other studies

Similar to our results which showed that TAPT decreased MACEs, TLR as well as TVR, Mohammad’s study published in 2010 also indicated that TAPT was more effective after drug eluting stents (DES) placement since it was associated with decreased TLR and MACEs in high risk patients [15]. Another example is Bangalore’s meta-analysis consisting of 41 trials conducted in 2014 [16]. This meta-analysis was also in favor of using cilostazol associated TAPT due to a significant reduction in both stent thrombosis and restenosis but, however, his study was not restricted to T2DM patients. Min’s study published in 2007 (including 23 % of the population with T2DM using TAPT and 29 % using DAPT) also concluded that the cilostazol based TAPT seemed to be more effective at preventing in-stent neointimal hyperplasia than a dual antiplatelet regimen [17]. Moreover, the results from the DECREASED registry showed that TAPT significantly reduced 12-month risks of stent thrombosis and MI after DES implantation compared with DAPT without any increase risk of bleeding complications [18]. In another meta-analysis, this time conducted by Sakurai, in 2013, TAPT was associated with significantly effective outcomes for TLR and TVR without any increase in MACEs [19]. A study by Kim published in 2011 including almost 30 % T2DM using TAPT, also supports our results showing that compared with high-dose clopidogrel in DAPT, adjunctive cilostazol significantly enhanced platelet inhibition and reduced the rate of high platelet reactivity showing that TAPT is apparently more effective than DAPT [20]. Moreover, the study by Douglas et al. which included 23 % of diabetic patients treated with TAPT and 28 % treated with DAPT showed that cilostazol-treated patients with oral hypoglycemic medications experienced a highly significant 63 % reduction in the risk of restenosis (*P* < 0.006) [21]. The study by Jeong et al. demonstrated that among his 30 % T2DM patients using TAPT and 13.3 % using the standard DAPT, the use of TAPT may achieve adequate inhibition of ADP-induced platelet aggregation to suppress the occurrence of major adverse cardiovascular events [22]. Finally, the CIDES trial which dealt with only T2DM patients showed that cilostazol was better than clopidogrel after drug eluting stents implantation in these patients. However, this study did not compare TAPT with DAPT; but instead, compared the clinical outcomes with the use of cilostazol and clopidogrel separately [23]. All of these studies show that TAPT is more effective and could safely be used as it is expected not to cause any increase in bleeding. In other words, apart from being more effective, TAPT is expected to be at least as safe as DAPT in T2DM patients.

However, even if many studies support our results, there are still a few studies whose results were completely different from our study. For example, the multicenter randomized trial conducted by Suh criticized the use of cilostazol as a component of the TAPT after stents implantation. But, the author accepted that his CILON-T trial had a different composition of patients and primary endpoints. The trial did not assess the beneficial role of cilostazol during PCI and included relatively low-risk patients. The author also precised that DECLARE DIABETES and DECLARE LONG studies which we have used in our meta-analysis had enrolled only high risk patients such as diabetes mellitus or long lesions. He also mentioned that his study was not designed to evaluate the post-treatment platelet reactivity [24]. Similarly, Jeon’s study conducted in 2010 demonstrated that there was no significant difference in the prevention of stent thrombosis between TAPT and DAPT groups but however, his study had a mixture of low and high risk patients [25]. Moreover, results from the HOST-ASSURE trial published in 2013 showed that the outcomes of both DAPT and TAPT were almost the same. TAPT was non inferior to double dose clopidogrel DAPT. However, the short follow-up period of 1 month could be a reason for this completely different result [26].

### Other anti-platelet agents

Apart from this cilostazol based TAPT, there are other anti-platelet agents which can be used after intracoronary stenting. For example, the early use of glycoprotein IIb/IIIa inhibitors can reduce the occurrence of death or myocardial infarction in patients with acute coronary syndromes who are not routinely scheduled for early revascularization. The event reduction is clinically most meaningful in patients at high risk of intracoronary thrombotic complications [27]. However, this drug can only be used for the short term in-hospital follow up. Moreover, the study by Geeganage’s showed that TAPT based on i.v GPIIb/IIIa inhibitors was associated with a higher bleeding complication than that of the standard DAPT; however, the diabetic status of these patients was unknown [28].

New drugs such as prasugrel and ticagrelor are gradually becoming known among the antiplatelet agents. However, these drugs are associated with a higher bleeding tendency and they lack the anti-restenosis property which is only possible with the use of cilostazol and hence, rendering this cilostazol based TAPT to be a better option for T2DM patients.

### Other potential benefits of cilostazol

Apart from being a part of TAPT, cilostazol alone has other potential indications. Studies have shown that cilostazol could reduce the symptoms of intermittent claudication [29, 30]. Cilostazol could also be used for the prevention of stroke in patients with non-cardio-embolic stroke [31]. Surprisingly, cilostazol has also shown to be beneficial to the heart. It can: increase the heart rate in patients with bradycardia, preventing ventricular fibrillation in patients suffering from Brugada syndrome, benefit patients with third degree atrio-ventricular block and could possibly act as a cardio-protective drug in patients with heart failure [32].

### Novelty in this study

Contrary to other studies, this meta-analysis has strictly been conducted on T2DM patients and has combined the adverse cardiovascular outcomes such as MACEs, TLR, TVR and stent thrombosis with platelet reactivity such as PRI and PRU; and has included all of them into one study. It can be said that TAPT could be a more promising drug therapy than the standard DAPT in T2DM patients after intracoronary stenting both in terms of effectiveness and safety.

### Limitations

Few limitations were as follow: First of all, because of the short follow up period and due to the limited study number and population size, the power of the analysis might be restricted to some extent. One study had a follow up of up to 2 years but this could not be considered for comparison because other studies did not have such long follow up periods. Secondly, due to the small population size, the results of this study may be affected to an extent. Also, the availability of new anti-platelet therapies such as prasugrel and ticagrelor could render TAPT useless in clinical practice. Unfortunately another limitation which restricts the use of this TAPT is that cilostazol is associated with a few adverse drug reactions such as rashes (RR 2.67; *P* = 0.01); gastrointestinal troubles (RR 1.76; *P* = 0.18); thrombocytopenia (RR 0.33; *P* = 0.50), hepatic dysfunction (RR 0.72; *P* = 0.67); palpitation (RR 6.18; *P* = 0.09) and headache (RR 3.56; *P* = 0.11). However, despite most of these adverse drug reactions being statistically insignificant in our study, drug discontinuation was significantly higher in the triple group (RR 3.75; *P* < 0.00001).

## Conclusion

Since MACEs have been significantly decreased in the triple group, TAPT appears to be more effective than DAPT in T2DM patients after intracoronary stenting. The insignificant differences in stent thrombosis, bleeding and most adverse drug reactions between these 2 groups result in TAPT to be almost as safe as DAPT in these diabetic patients.
